# Altered Neural Activity Associated with Mindfulness during Nociception: A Systematic Review of Functional MRI

**DOI:** 10.3390/brainsci6020014

**Published:** 2016-04-19

**Authors:** Elena Bilevicius, Tiffany A. Kolesar, Jennifer Kornelsen

**Affiliations:** 1 Department of Physiology and Pathophysiology, The University of Manitoba, Winnipeg, MB R3E 0J9, Canada; bilevice@myumanitoba.ca (E.B.); Tiffany.Kolesar@umanitoba.ca (T.A.K.); 2 Department of Radiology, The University of Manitoba, Winnipeg, MB R3T 2N2, Canada; 3 St. Boniface Hospital Research, Winnipeg, MB R2H 2A6, Canada

**Keywords:** systematic review, mindfulness, nociception, pain, functional MRI

## Abstract

Objective: To assess the neural activity associated with mindfulness-based alterations of pain perception. Methods: The Cochrane Central, EMBASE, Ovid Medline, PsycINFO, Scopus, and Web of Science databases were searched on 2 February 2016. Titles, abstracts, and full-text articles were independently screened by two reviewers. Data were independently extracted from records that included topics of functional neuroimaging, pain, and mindfulness interventions. Results: The literature search produced 946 total records, of which five met the inclusion criteria. Records reported pain in terms of anticipation (*n* = 2), unpleasantness (*n* = 5), and intensity (*n* = 5), and how mindfulness conditions altered the neural activity during noxious stimulation accordingly. Conclusions: Although the studies were inconsistent in relating pain components to neural activity, in general, mindfulness was able to reduce pain anticipation and unpleasantness ratings, as well as alter the corresponding neural activity. The major neural underpinnings of mindfulness-based pain reduction consisted of altered activity in the anterior cingulate cortex, insula, and dorsolateral prefrontal cortex.

## 1. Introduction

Pain is defined as “an unpleasant sensory and emotional experience associated with actual or potential tissue damage, or described in terms of such damage” [1]. Although pain is typically thought of as a singular concept, it is a complex process that is comprised of three key components: sensory (intensity, location, duration), cognitive (appraisal, attention), and affective-motivational (unpleasantness, desire to escape) [2]. The sensory component of pain activates somatosensory cortex [3,4], the cognitive component activates the prefrontal cortex [5,6], and pain unpleasantness is associated with increased activity in the anterior cingulate cortex (ACC) and insula [3,4,6,7].

Pain is often managed with pharmaceutical interventions; however, recent evidence suggests that mindfulness, a relatively risk-free alternative, is successful in attenuating anticipation [8,9,10], and unpleasantness of pain [8,9,10,11,12,13], and can alter pain intensity [8,9,11,12,14]. Mindfulness is defined as a mental state achieved by purposeful awareness on the present moment with an accepting and nonjudgmental stance [15,16]. This mental state has been documented to produce both psychological [15,17,18] and physiological [19,20,21,22] benefits for healthy and patient populations. Although mindfulness is said to engage multiple brain regions that mitigate the subjective appraisals of pain, the exact underlying neural mechanisms of mindfulness-based pain reduction are not fully understood. In the current systematic review, studies in which functional magnetic resonance imaging (fMRI) has been used to identify the brain regions associated with mindfulness-based pain reduction have been compiled [8,9,11,12,23].

The purpose of the present research is to gather all of the relevant literature to determine which brain regions are altered by mindfulness in response to pain. To this end, a systematic review was conducted to evaluate and summarize the existing fMRI and positron emission tomography (PET) investigations of how mindfulness attenuates pain. The result of this research will shed light on the success of mindfulness as an effective, risk-free option for the attenuation of the multifaceted process of pain.

## 2. Materials and Methods

### 2.1. Literature Search and Selection Criteria

A systematic search of the literature was conducted on 2 February, 2016, in consultation with a medical librarian. Each of the Cochrane Register of Controlled Trials (Central), EMBASE, Ovid Medline, PsycINFO, Scopus, and Web of Science databases were searched from inception. The search identified abstracts that included Medical Subject Headings, text, and keywords consistent with three major ideas: (1) functional neuroimaging (including PET and fMRI); (2) pain; and (3) mindfulness. Language and publication dates were unrestricted.

All titles and abstracts were reviewed independently by two reviewers (E.B. and T.A.K.). Abstracts were included for further analysis if selected by either party. The full text articles were independently reviewed by both reviewers as well, but were included or rejected once a consensus was reached. After the initial search was completed, the reference lists of the included articles were searched to determine if any additional appropriate papers could be identified.

Original research papers that included a mindfulness intervention or participant group were initially included if functional neuroimaging was used during the application of a noxious stimulus, or in patients with acute or chronic pain. Full-text articles were retained if they included a variation of the following definition: mindfulness is a mental state achieved by awareness on the present moment with an accepting and nonjudgmental stance. Subsequent full-text articles were excluded if they did not explicitly provide a definition of mindfulness.

### 2.2. Data Extraction and Synthesis

Two reviewers independently extracted data from the included articles, including publication year, population, number of participants, type of mindfulness training and mindfulness measure, type of noxious stimulation, pain ratings, region of stimulus application, and neural activity. Additionally, demographic data including participant sex distribution, mean age, handedness, and location of data collection were extracted.

### 2.3. Assessment of Study Consistency

Inconsistencies were qualitatively assessed across the studies to determine to what extent the results could be generalized.

## 3. Results

### 3.1. Identification of Studies

**Figure 1:**
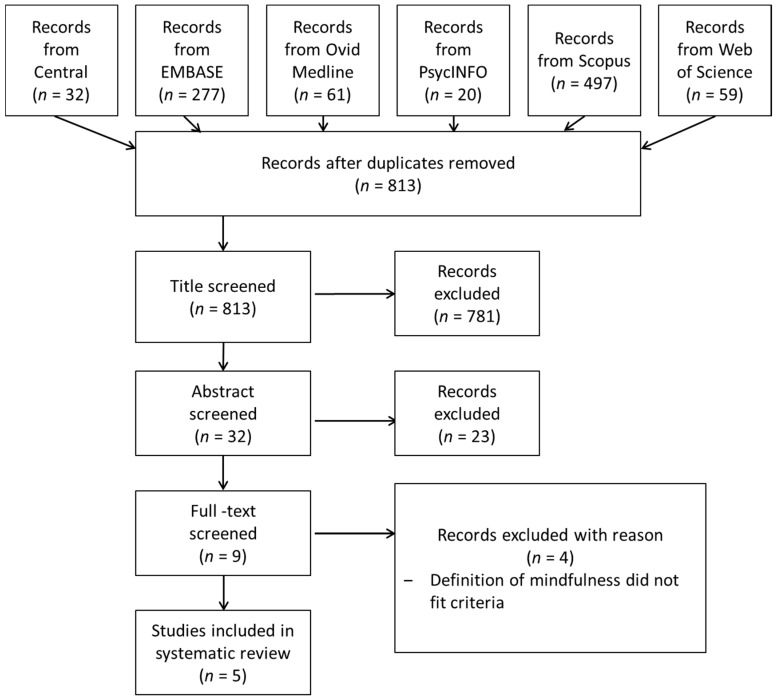
Flowchart of procedure. Central indicates the Cochrane Central Register of Controlled Trials database.

The search strategy produced 946 total citations: 32 from Central, 277 from EMBASE, 61 from Ovid Medline, 20 from PsycINFO, 497 from Scopus, and 59 from Web of Science (see Figure 1). A total of 133 duplicates were removed, leaving 813 records to examine. After screening the titles, 781 of these records were excluded, resulting in 32 remaining abstracts. All but nine records were excluded after reading the abstracts, and of these, only five relevant articles were retained. The four records removed after reading the full-text articles were excluded, as they did not provide an explicit definition of mindfulness. No additional references were obtained upon examination of the reference lists.

### 3.2. Details of Included Studies

Details of the included studies are presented in Table 1. Studies examining how mindfulness alters pain intensity, anticipation, and unpleasantness with fMRI ranged in publication date from 2011 to 2015. No records were found that measured pain perception during a mindfulness intervention while using PET as the neuroimaging technique. Upon reviewing the literature, there was a lack of research conducted with acute or chronic pain populations; experimentally-induced pain was measured in all of the included studies. Thermal nociception was used in the majority of studies (*n* = 4) [9,11,12,23], while electrical stimulation was used in the other study [8]. The measure of mindfulness used varied between the studies. Three studies used the Freiburg Mindfulness Inventory (FMI) [24] to measure state levels of mindfulness [8,11,12], while the other two used non-validated self-report measures. Specifically, one study used a questionnaire developed to assess meditative history [23], and the other relied on hours or years of formal mindfulness meditation training as the measure of mindfulness [9].

Mean participant age for mindfulness practitioners and healthy controls were reported for all five studies. The average participant age in the studies was 36.80 years [8,9,11,12,23]. The number of participants ranged from 15 to 75, with an equal number of meditators and healthy controls when two population groups were used [8,9,11,12,23]. Sex was reported in all of the studies with approximately 60% being males and 40% being females. Handedness was only reported in three studies: 16 out of 17 were right handed in one study [8] and all participants were right handed in the other two [11,12].

All of the studies included a meditation or mindfulness group, measured mindfulness in different ways (FMI [8,11,12], years of mindfulness practice [23], and hours of training [9]), and applied painful stimulation while in the MRI scanner (see Table 1). In the paper by Gard and colleagues [8], transcutaneous electrical stimulation was randomly delivered to the forearm in both mindfulness and baseline conditions, using mixed block design [8]. In the mindfulness condition, participants were instructed to act mindfully, specifically focusing their attention on the location beneath the electrode while being aware and accepting of the noxious stimulus [8]. Both mindful and control conditions were used in the study conducted by Grant and colleagues [23]; however, only baseline levels were reported for both groups, *i.e.*, the participants in the mindfulness condition were explicitly told not to meditate. Thermal stimuli were preceded with audible sounds in order to prepare and bring awareness to which stimulus was impending [23]. In the study by Lutz *et al.* [9], an experimental block design was used that involved focused attention (FA) or open presence (OP) meditation before either a hot or warm thermal stimulus was applied to the forearm in experienced meditators and controls [9]. FA practice involves purposeful attention to a specific object, whereas OP practice includes the openness and acceptance of mindfulness [9]. Participants were informed prior to the onset of noxious stimulation, and the neural activity occurring during this time was considered to be activation during anticipation of pain. Both of the remaining papers written by Zeidan and colleagues [11,12] involved pre- and post-MRI scans and experimental sessions with inexperienced meditators. In the earlier of the two studies [11], the first session was an attention to breath (ATB) task, which was a way for participants to become familiar with meditation training. At this time, participants were explicitly asked to begin meditating through an ATB task before a series of hot and neutral stimuli were presented on the calf in a block design experiment [11]. Following this session, participants engaged in a four-day mindful meditation intervention, after which they entered the magnet again. The stimuli were applied randomly as the participants were instructed to begin their meditation [11]. Finally, Zeidan and colleagues [12] trained inexperienced meditators in mindfulness, sham-mindfulness, placebo, and control groups to make a neural distinction between them. This took place in multiple sessions, where true mindfulness practice, sham, or unrelated instruction was presented before either a hot or neutral stimulus was applied to the calf. Based on these methodologies, it is evident that all of the included studies were fairly consistent, which resulted in similar findings of mindfulness-based pain reductions.

### 3.3. Behavioural Data

The behavioural data, when collected, was fairly consistent across the studies. When anticipation of pain data was collected, participants in mindfulness conditions reported lower anticipation of pain than controls during meditation [8,9]. Additionally, a reduction in pain unpleasantness scores was typically reported in the mindfulness conditions [8,9,11,12]. Unpleasantness ratings did not differ in one study, but this is likely because ratings were for baseline conditions of both mindful and control conditions (*i.e.*, meditators were not meditating) [23]. Pain intensity scores remained constant in two studies [8,9], and decreased in two others [11,12]. In the other study, pain intensity was rated initially by control and mindfulness groups to determine a moderate level of pain—the mindfulness condition required a higher temperature to reach the same intensity level [23].

### 3.4. Neural Activity

#### 3.4.1. Identification of Neural Activity

Three of the studies reported voxel counts in addition to coordinates and associated statistics [8,9,23]. The specific Brodmann area was also identified in one of these three studies [8]. The two remaining studies reported z-scores, coordinates, and their corresponding regions only [11,12]. However, all five studies reported significant coordinates for the reported neural activity, allowing the altered brain regions to be identified for every study. In this way, the results were comparable from study to study.

#### 3.4.2. Trends in General Pain and Pain Intensity

All five studies reported altered activity within the insula in mindfulness practitioners compared to controls, in response to noxious stimulation *versus* baseline [8,9,11,12,23]; see Table 2. Similarly, all studies demonstrated modulated activity in the ACC as it related to the pain intensity [11,12,23] or if a mindful >control contrast produced changes in both unpleasantness scores and neural activity [8,9,23]. The results of the thalamus were mixed, with one study demonstrating an increase in activity during mindfulness [23] and another two demonstrating a decrease in activity compared to the healthy control group [11,12]. Finally, the studies reported lateral [8], or dorsolateral prefrontal cortex (dlPFC) [12,23] deactivation in mindfulness practitioners compared to healthy controls while Gard and colleagues [8] reported greater ventromedial PFC activity for mindfulness conditions. Interestingly, the dlPFC is a major component of the Central Executive Network (CEN), an example of a resting state network [25]. Resting state networks are comprised of functionally connected regions that activate in synchronous oscillations when the brain is at rest [26]. The CEN is important when performing cognitive tasks, such as decision-making, and is associated with the control of processing of information [25,27]. Importantly, the dlPFC is also involved in modulating pain processing [28].

In order to delineate the components of pain, many of the studies separated pain into two or three subcategories. Unfortunately, although pain intensity ratings were commonly reported in the studies, these ratings were often not directly related to the neural activity observed. Decreased general or intensity pain ratings were associated with increased activity in the insula [8,9,11,12,23] and ACC [9,11,12,23]. In contrast, in a resting condition, meditation experience was correlated with decreased pain-related activity in the insula and ACC [23].

In the paper by Zeidan and colleagues [12], both the placebo and sham-mindfulness group had different results than the mindfulness meditation group. Specifically, instead of activations in the insula and ACC in response to pain stimulation, the placebo group reported deactivations in the insula and the sham group in the ACC [12]. This demonstrates a difference in neural activation patterns between true mindfulness groups and sham or placebo groups.

#### 3.4.3. Anticipation of Pain and Mindfulness

To identify the cognitive component of pain, two research groups measured how neural activity associated with the anticipation of a painful stimulus varied between the mindfulness and healthy control groups [8,9]. Of note, in the study by Gard and colleagues [8], an interaction was observed in the rostral ACC and ventromedial PFC for pain anticipation anxiety during the application of mindfulness when the meditation group was compared with controls. The researchers believe that mindfulness modulates pain anticipation using a unique mechanism not shared by other pain regulation practices. Based on this theory, the lateral PFC acts to increase activity in the rostral ACC, which in turn decreases the activation in typical pain-processing regions [8,29].

Lutz and colleagues [9] altered their question regarding anticipation of pain during mindfulness, interested instead on comparing the neural activity in the time after the cue of the noxious stimulus prior to actual stimulation, rather than behavioural measures. These researchers considered regions-of-interest including the middle to posterior insula, secondary somatosensory cortex (SII), and the midcingulate cortex (MCC). According to expectations, Lutz and colleagues [9] found that baseline activity was greater in two clusters (left anterior insula and anterior MCC) for controls compared to the meditation practitioners. In addition to these results, the researchers found that mindfulness practitioners habituated to pain and anticipation anxiety faster than controls, specifically in the amygdala [9]. Interestingly, activity in the MCC (associated with appraisal of pain) attenuated faster when baseline amygdala activity (implicated in anxiety) was low [9].

#### 3.4.4. Unpleasantness of Pain and Mindfulness

The affective component of pain, or unpleasantness, was the most commonly reported component and had overlapping results with those of pain intensity. The researchers did not normally correlate neural activity with unpleasantness; however, increased activity was found in ACC [8,9,11,12,23] and insula in mindfulness participants [8,9,11,12,23], along with decreased pain unpleasantness ratings in four of the five studies [8,9,11,12]. As expected, based on these results, Gard and colleagues [8] observed a negative correlation between pain unpleasantness, and activity in the ACC. This consistency suggests that mindfulness may play an important role in controlling the affective component of pain by altering the activity of the insula and ACC. Further, because Zeidan and colleagues [12] used a pre- and post-study design, the causality of mindfulness on neural regulation of pain can be implied. As discussed by Lutz and colleagues [9], the anterior insula and cingulate cortex are parts of the salience network (SN) in the brain [25,30]. The SN, like the CEN, is a functionally connected resting state network and works to integrate sensory, cognitive, and emotional information—this finding is not surprising given that these three components sum to create the whole phenomenon of pain [30].

Participants in the mindfulness condition of one study also had increased activity in SII which correlated with affective pain ratings, likely resulting from the instructions explicitly guiding them to focus on the pain and its location [8]. Grant and colleagues [23] also reported increased activity in SII, but in response to general pain. Zeidan and colleagues [11] observed decreased activity in primary somatosensory cortex (SI) in the mindfulness condition, but did not indicate which component of pain was being measured. Participants in these conditions were concentrating on the sensory component of pain (*i.e.*, intensity and location), which may have diverted resources away from the emotional (*i.e.*, unpleasant) aspect of pain. These results indicate that mindfulness may influence areas of the brain in different ways in an attempt to attenuate the unpleasantness of pain [9].

### 3.5. Study Consistency

Among the five included studies, the methodology varied mildly. In data acquisition, field strength of the magnet varied amongst the studies, either 1.5 [8,11] or 3 [9,12,23] Tesla. The preprocessing and analysis software differed between the studies. Software programs included BrainVoyager QX 1.10.4 [8], BrainVoyager QX [23], AFNI [9], and FSL [11,12]. In the statistical analyses of the papers, all five implemented a whole-brain random effects general linear model analysis [8,9,11,12,23]. Lutz and colleagues also carried out a region of interest analysis of task-based findings to investigate their neural habituation hypothesis [9]. Contrasts of interest were then executed, typically comparing the mindfulness condition to the baseline in both the mindfulness and control conditions. Four of the five reports used a significance cutoff of *p* < 0.05 [8,9,11,12], and the remaining report used a more stringent significance value of *p* < 0.01 [23]. The Monte Carlo Simulation was the favoured method for correction for multiple comparisons (*n* = 3) [8,9,23]. Most of the studies (*n* = 3) warped their data into Talairach space [8,9,23], while one used MNI [12] and the other did not report which standardized space was used [11]. Although some studies reported voxel counts in addition to significance values [8,9,23], others included only z-scores [11,12]. Three studies reported the use of peak voxel coordinates [8,9,23], with one of these studies also reporting centre of mass [23]. The remaining two reports did not identify which coordinates were used [11,12]. Despite the consistent methodologies of the reported studies as they all included investigations of pain in fMRI and mindfulness, they do not all compartmentalize pain the same way. Some separated pain into anticipation, unpleasantness, and intensity, while others viewed pain more generally. Due to this difference, the authors concluded that although mindfulness does reduce pain and alter the associated neurological activity, it cannot be pinpointed to a specific component of pain that it is acting on.

## 4. Discussions

The results were fairly consistent across a number of different factors in each of the five studies. First, mindfulness reduced the affective experience of experimentally-induced pain, as indicated by reduced affective pain ratings during mindfulness interventions [8,9,11,12]. Anticipation ratings were also decreased during the mindfulness intervention in both studies that reported these values [8,9]. Interestingly, pain intensity ratings decreased or remained unchanged in the mindfulness interventions; this uncertainty is also reported in the literature [31].

Although the consistency of the results may at first seem puzzling—that is, activity in pain related regions (ACC and insula) increased during meditation—they can be understood within the broader context of neural function. In reality, the brain regions making up the “pain matrix”, namely the ACC, insula, SI and SII, PFC, and thalamus are not solely used for pain perception [32]. Rather, structures within this network have multiple broader roles. For example, the ACC and insula have roles in emotion and attention [33], and dlPFC is important for working memory [34] and moral decisions [35]. Additionally, an electroencephalography (EEG) study showed that people scoring as more sensitive on the Behavioural Inhibition Scale showed greater neural activity in their right dlPFC [36]. This scale indicates a person’s reactivity to threat [36]; a person experiencing mindfulness in the currently reviewed studies removed judgment of the present surroundings, thus they are reducing their reactivity to threat, which may account for the reduced dlPFC activity seen in three of the studies [8,12,23]; see Table 2. Following from this perspective, increased activity in ACC and insular regions during mindfulness interventions can be explained by the increased attention that the mindful individual is placing on the present moment. In light of a recent real-time fMRI neurofeedback meta-analysis, it was suggested that the insula is a key active region involved with self-regulation [37]. This is another possible mechanism explaining the activity associated with mindfulness in the reviewed studies as people trained in mindfulness actively direct their attentional resources back to the present if and when it drifts. Thus, increased neural activity in these “pain regions” does not necessarily indicate that the participant is experiencing greater pain, which is corroborated by their decreased pain ratings, but instead may indicate their increased attentional resources as they enter a mindful state. Additionally, the reduced dlPFC activity may be related to the reduction in judgment of the surroundings.

From a network perspective, these brain regions are also represented in the SN and CEN. Increased activity was commonly observed in the key structures of the SN (insula and ACC) while the dlPFC is a major component of the CEN. These results indicate a possible mechanism of pain attenuation via upregulation of the SN and downregulation of the CEN. Further research needs to be conducted using resting state fMRI in a mindfulness condition to investigate whether or not functional connectivity supports these observations.

This systematic review incorporates only five studies, each comprised of similar results showing that mindfulness functionally alters brain activity in response to experimentally-induced pain. Unfortunately, the results from these fMRI studies cannot be generalized to those living with chronic pain, although evidence suggests that mindfulness is an effective strategy for coping with chronic pain [38,39]. However, there have been studies that use EEG to assess how mindfulness alters brain activity in the anticipation of pain. In 2013, Brown and colleagues [40] enrolled half of their chronic pain participants in a mindfulness-based pain management program; the remaining chronic-pain participants comprised the no-treatment group. Due to the difficulties manipulating chronic pain experimentally, acute thermal pain was administered with a laser in both groups. In the mindfulness-intervention group, event-related potentials revealed increased activation in the dlPFC and this was interpreted as a potential emotional regulation strategy. This may suggest that mindfulness alters the neural mechanisms of anticipation of acute pain in chronic pain patients by increasing the activity in areas of the brain that are associated with emotion-regulation, which echoes the fundamental principles of mindfulness. In contrast to this research in chronic pain patients, the present systematic review found decreased dlPFC activity, which may suggest differential mindfulness-based pain reduction mechanisms in experimentally-induced and chronic pain.

Clinical trials and meta-analyses have investigated the success of mindfulness-based interventions for chronic pain populations. In a meta-analysis from 2014 [41], no specific effect from mindfulness was found to reduce pain intensity in chronic pain patients, but there were significant reductions in both psychological variables such as anxiety and depression. However, a review by Reiner and colleagues [31] demonstrated that half of the studies reviewed reported decreased pain intensity ratings in the mindfulness group compared to the control groups. Following this work, a recently published randomized controlled trial found that mindfulness based stress reduction enhanced the pain management skills in individuals with nonspecific chronic pain [42]. Further, although they indicated the level of pain did not change, improved pain acceptance and coping were present both immediately following the intervention and 6 months post-intervention. Clearly, there is disparity in the literature about the effectiveness of mindfulness to relieve pain intensity ratings in various chronic pain patients, as observed in our own experimental pain findings. As chronic pain is difficult to manipulate experimentally, it is important to understand the neuroscience of mindfulness in chronic pain populations. Elucidating the mechanism of mindfulness on pain modulation will provide both basic science implications for future research, and practical expectations and implications for potential patients. Future neuroimaging research should investigate the neural correlates of mindfulness-based pain interventions in chronic pain populations.

The present study is limited by the strict use of the mindfulness definition that was enforced as an exclusionary criterion. As a result, papers that included similar methodology to the included papers may have been excluded simply due to ambiguity, or lack of an operational definition. This key criterion likely reduced the variability of neural activity patterns that may have otherwise been obtained. The use of this definition ensured that the present studies measured the same or highly similar mindfulness constructs.

It is worth mentioning that a review paper by Nakata and colleagues (2014) also investigated the role of mindfulness in pain reduction, but had a broader definition of mindfulness [43]. By systematically reviewing the literature, we have come to similar conclusions regarding the brain regions involved in mindfulness-based pain reductions. By limiting the definition of mindfulness and providing a detailed account of the relevant methodologies and results, we were able to provide a clearer picture of how mindfulness, as we have defined it, alters the different aspects of pain.

## 5. Conclusions

In summary, from the collection of studies reviewed, it appears that mindfulness practitioners reduce their affective experience of experimentally-induced pain while reductions of pain intensity ratings are less consistent. The neural mechanisms behind this pain alteration appear to be linked to upregulation of brain regions that are key nodes of the SN and downregulation in nodes of the CEN. Future research is required to determine if these mechanisms are common in acute and chronic pain, as well as whether people high in mindfulness have altered functional connectivity in these resting state networks.

## Figures and Tables

**Table 1:** Characteristics of the included studies.

First Author (Year)	Population	(N_M_/N_C_) ^1^	Training	Mindfulness Measure ^2^	Stimulus (Time) ^3^	Initial Pain Ratings ^4^	Stimulation Region
Gard (2012) [8]	Mindfulness Practitioners	N_M_ = 17 N_C_ = 17	Vispassana	FMI	Elec: 103+ V, 833 Hz (0.1 s)	I: ~4.6/10 U: ~4.4/10 A: ~3.1/10	Left lower arm
Grant (2011) [23]	Meditation Practitioners	N_M_ = 13 N_C_ = 13	Zen Mediation	Years of practice	Therm: Av. Zen 49.9 °C Av. Cntrl 47.9 °C (10 s)	I: 6–7/10	Lateral, posterior left calf
Lutz (2013) [9]	Meditation Practitioners	N_M_ = 14 N_C_ = 14	Nyingma, Kagyu	>10,000 h of training	Therm: Av. 48.1 °C (10 s)	P: 8/10	Inside of left forearm
Zeidan (2011) [11]	Healthy Controls	N_C_ = 15	4 days of training	FMI	Therm: 49 °C (12 s)	I: 4.8/10 U: 4.8/10	Posterior right calf
Zeidan (2015) [12]	Healthy Controls	N_C_ = 75	4 days of training	FMI	Therm: 49 °C (12 s)	I: 4.8/10 U: 5/10	Posterior right calf

^1^ *N*_M_ = number of participants in mindfulness conditions; *N*_C_ = number of control participants. ^2^ FMI = Freiburg Mindfulness Inventory. ^3^ Elec = electrical simulation; Therm = thermal stimulation. ^4^ Pain Ratings: I = intensity, U = unpleasantness; A = anticipation, P = general pain rating. Stimulation temperature was consistent across participants, or reported as an average (Av.). The study by Grant [23] reported average temperatures for experienced meditators (Av. Zen = average temperature used in meditator condition; Av. Cntrl = average temperature used in control condition). Pain ratings are for the mindfulness conditions and are the initial pain ratings obtained prior to neuroimaging.

**Table 2:** Selected neural activity reported in included studies.

Brain Regions	Pain Dimension	Author	Contrast	Component	*t*-Value/Z Score (*p*-Value)
**Anterior Cingulate Cortex**					
	General	Lutz	Mf > H	Anterior Middle	*t* = 3.9 (*p* > 0.005)
	General	Grant	Mf > H	Dorsal	*t* = 3.64 (*p* > 0.001)
	Intensity	Grant	Mf > H	Dorsal	*t* = 2.22 (*p* = 0.04)
	Intensity	Zeidan (2011)	Mf > H	Anterior	Z = 5.35 Z = 5.30 Z = 5.29
	Intensity	Zeidan (2015)	Mf > H	Subgenual	Z = 5.61
	Anticipation	Lutz	Mf > H	Bilateral	*t* = 3.1 (*p* < 0.005) *t* = −4 (*p* < 0.0005)
	Anticipation ^1^	Gard	Mf > H	Rostral/VM/mFG	*t* = 2.8 (*p* = 0.009)
**Insula**					
	General	Grant	Mf > H	Posterior/Thalamus/BG	*t* = 3.61 (*p* > 0.001) *t* = 4.61 (*p* > 0.001)
	General	Lutz	Mf > H	Anterior	*t* = 3.4 (*p* < 0.005)
	Intensity	Grant	Mf > H	Anterior Middle/iFG/Thalamus/BG	*t* = 2.44 (*p* = 0.02) *t* = 2.46 (*p* = 0.02) *t* = 2.98 (*p* = 0.007) *t* = 3.29 (*p* = 0.003)
	Intensity	Zeidan (2011)	Mf > H	Anterior	Z = 3.04
	Intensity	Zeidan (2015)	Mf > H	Anterior	Z = 4.05
	Anticipation Unpleasantness	Lutz	Mf > H	Anterior Posterior	*t* = −3.8 (*p* < 0.005) *t* = −4.5 (*p* < 0.0005)
	Unpleasantness	Zeidan (2011)	Mf > H	Anterior	Z = 2.73
	Unpleasantness ^2^	Gard	Mf > H	/Somatosensory	*t* = 3.59 (*p* = 0.001)
**Somatosensory I**					
	General	Zeidan (2015)	Mf > H		Z = 3.07
**Somatosensory II**					
	General	Grant	Mf > H	/Parietal Operculum	*t* = 4.39 (*p* < 0.001)
	Unpleasantness ^2^	Gard	Mf > H	/Insula	*t* = 3.59 (*p* = 0.001)
**Prefrontal Cortex**					
	General	Gard	H > Mf	Lateral/mFG	*t* = 4.03 (*p* < 0.001) *t* = 3.87 (*p* < 0.001)
	General	Zeidan (2015)	H > Mf	Dorsolateral	Z = 4.10 Z = 3.82 Z = 4.65
	Intensity	Grant	H > Mf	Dorsolateral	*t* = 2.22 (*p* = 0.04) *t* = 2.84 (*p* = 0.009) *t* = 3.54 (*p* = 0.002)
	Anticipation ^1^	Gard	Mf > H	VM/mFG/rACC	*t* = 2.8 (*p* = 0.009)
**Thalamus**					
	General	Grant	Mf > H	/BG/Insula	*t* = 4.61 (*p* > 0.001)
	Intensity			/BG/Insula	*t* = 3.29 (*p* = 0.003)
	Unpleasantness	Zeidan (2011)	H > Mf		Z = 6.74 Z = 3.12
	General	Zeidan (2015)	H > Mf		Z = 5.75 Z = 4.63

^1,^ ^2^ indicate two different repeated clusters, for completeness and ease of reading. Note that neural activity is as reported in terms of pain dimension, but the text describes how this activity changes concurrently with, e.g., unpleasantness ratings (VM = ventromedial; m/iFG = middle/inferior frontal gyrus; BG = basal ganglia; H = healthy controls; Mf = mindfulness condition).

## Abbreviations

The following abbreviations are used in this manuscript:

ACC	anterior cingulate cortex
ATB	attention to breath
CEN	central executive network
dlPFC	dorsolateral prefrontal cortex
FA	focused attention
FMI	Freiburg Mindfulness Inventory
fMRI	functional Magnetic Resonance Imaging
MCC	midcingulate cortex
OP	open presence
PET	Positron Emission Tomography
SI	primary somatosensory cortex
SII	secondary somatosensory cortex
SN	salience network
